# Stable Isotope Ratio Analysis for the Geographic Origin Discrimination of Greek Beans “Gigantes-Elefantes” (*Phaseolus coccineus* L.)

**DOI:** 10.3390/foods13132107

**Published:** 2024-07-02

**Authors:** Anna-Akrivi Thomatou, Eleni C. Mazarakioti, Anastasios Zotos, Efthimios Kokkotos, Achilleas Kontogeorgos, Angelos Patakas, Athanasios Ladavos

**Affiliations:** 1 Department of Food Science & Technology, University of Patras, 30100 Agrinio, Greece; 2 Department of Sustainable Agriculture, University of Patras, 30100 Agrinio, Greece; 3 Department of Agriculture, International Hellenic University, 57001 Thessaloniki, Greece

**Keywords:** geographical origin, authenticity, beans, isotope ratio mass spectrometry (IRMS)

## Abstract

Adulteration of high-value agricultural products is a critical issue worldwide for consumers and industries. Discrimination of the geographical origin can verify food authenticity by reducing risk and detecting adulteration. Between agricultural products, beans are a very important crop cultivated worldwide that provides food rich in iron and vitamins, especially for people in third-world countries. The aim of this study is the construction of a map of the locally characteristic isotopic fingerprint of giant beans, “Fasolia Gigantes-Elefantes PGI”, a Protected Geographical Indication product cultivated in the region of Kastoria and Prespes, Western Macedonia, Greece, with the ultimate goal of the discrimination of beans from the two areas. In total, 160 samples were collected from different fields in the Prespes region and 120 samples from Kastoria during each cultivation period (2020–2021 and 2021–2022). The light element (C, N, and S) isotope ratios were measured using Isotope Ratio Mass Spectrometry (IRMS), and the results obtained were analyzed using chemometric techniques, including a one-way ANOVA and Binomial logistic regression. The mean values from the one-way ANOVA were *δ*^15^N_AIR_ = 1.875‰, *δ*^13^C_V-PDB_ = −25.483‰, and *δ*^34^S_V-CDT_ = 4.779‰ for Kastoria and *δ*^15^N_AIR_ = 1.654‰, *δ*^13^C_V-PDB_ = −25.928‰, and *δ*^34^S_V-CDT_ = −0.174‰ for Prespes, and showed that stable isotope ratios of C and S were statistically different for the areas studied while the Binomial logistic regression analysis that followed correctly classified more than 78% of the samples.

## 1. Introduction

In our days, food authentication is a subject of matter in the food industry. Agricultural products with geographical marks are more tempting and attractive for consumers, increasing their interest and impelling them to pay extra money for these indications. Simultaneously, as the prices of these products increase, financial gain leads to adulteration incidents [1,2].

The European Union was forced to describe food origins, and for this reason, it has published a Traceability Regulation (178/2002/EC) that defined “food and feed traceability”. Furthermore, in 1992, the European Union legislated the Protected Designations of Origin (PDO) and Protected Geographical Indication (PGI) schemes (Regulation 2081/92/EEC), which was followed by Regulation 510/2006/EEC and 1151/2012/EU that are European laws against mislabeling [1,3,4].

A problem that many consumers frequently face is the adulteration of high-quality legumes with ones of poor quality that have the same appearance [5]. Legumes are used in significant quantities in the human diet because they are regarded as a necessary nutrient origin [6,7,8]. The common bean (*Phaseolus vulgaris* L.) is one of the most important dry legumes in the legumes listed by the FAO, including up to 117 species. Dry beans are an essential source of protein, iron, minerals, vitamins, energy, and dietary fiber for many people worldwide, especially in third-world countries where people are suffering from malnutrition [8]. Furthermore, the consumption of beans has many advantages that are strongly related to health improvements, such as the prevention of cardiovascular disease, obesity, decrease in serum cholesterol and hyperglycemia, and the prevention of colon, breast, and prostate cancer [9,10,11,12,13,14,15,16,17]. After *Phaseolus vulgaris*, the bean species that is very important worldwide is the runner bean (*Phaseolus coccineus* L.). In Greece, one of the runner bean species that is cultivated is Gigantes-Elefantes, which are large white-seeded beans with white blossoms and continuous flowering. Due to its high quality, it is grown under protective law, and it is a Protected Geographical Indication (PGI) product. The product is cultivated in three regions in the north of Greece: Kato Nevrokopi (L 15/21-1-1998, EC No 134/1998), Kastoria (L 203/12-8-2003, EC No 1428/2003), and Florina (Prespes) (L 202/18-7-1998, EC No 1549/1998).

In many cases, the creation of a trustable differentiation system is essential to check the origin of food products. Traceability methods are a very useful tool for the determination of the geographical origin of foodstuff. Although various studies have been published worldwide that concern the application of these methods to different food products, little data exists about the application of these techniques for the discrimination of legume origins. Laursen et al. (2011) [18] investigated the multi-elemental composition of faba beans (*Vicia faba* L.) using Inductively Coupled Plasma Optical Emission Spectrometry (ICP-OES) and Mass Spectrometry (ICP-MS). More specifically, discrimination was attained with multi-elemental fingerprints consisting of 14 elements, which was further reinforced when analyzing 25 elements with semiquantitative ICP-MS. Longobardi et al. (2015) [19] used Isotope Ratio Mass Spectrometry (IRMS) for the discrimination of the geographical origin of lentils *(Lens Culinaris* L.) from Italy and Canada. Samples were analyzed to determine the stable isotope ratio of δ^13^C, δ^15^N, δ^34^S, δ^18^O, and δ^2^H, showing different values for the two geographical areas for all parameters except δ^15^N. Ganopoulos et al. (2012a) [20] used a High-Resolution Melting (HRM) application coupled with DNA barcoding (Bar-HRM) for the measurement of the adulteration of “Fava Santorinis” species. Bar-HRM is a rapid and precise method that yielded good results in the discrimination of PDO “Fava Santorinis” from other food products called “fava”. They also used HRM analysis on microsatellite markers for the authentication of “Plake Megalosperma Prespon”, a common bean variety cultivated in Northern and Central Greece [21]. This combined approach proved to be a helpful technique for the discrimination of different bean varieties. Fan and Beta (2017) [22] determined the geographical origin of common beans (*Phaseolus vulgaris* L.) using phenolic profiles and antioxidant activity. More specifically, samples from areas of higher altitudes were found to have a higher content of phenolic compounds and antioxidant activities than those from areas with lower altitudes, concluding that this method is appropriate for samples from different areas. Santos et al. (2013) [23] used Inductively Coupled Plasma Optical Emission Spectrometry (ICP-OES) for the determination of the geographical origin of common beans. After optimizing different experimental factors to obtain good accuracy, samples were analyzed in order to discriminate according to their geographical origin.

The aim of this study was the discrimination of “Gigantes-Elefantes” beans derived from growing areas of Kastoria and Prespes using the stable isotope ratio methodology and the construction of a map of the locally characteristic isotopic fingerprint of giant beans with the ultimate goal of the protection of those of similar products produced in other countries.

## 2. Materials and Methods

### 2.1. Sampling

**Figure 1:**
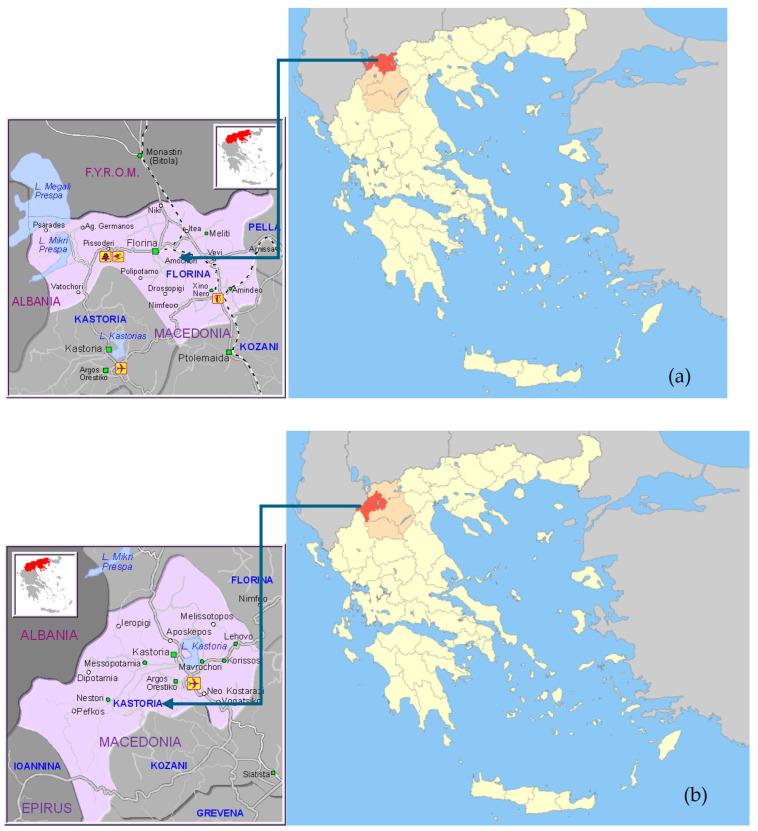
The areas cultivating the examined Giant beans samples. ((**a**) Prespes, Florina; (**b**) Kastoria).

Sampling was performed in two cultivation periods in Prespes, Florina (Figure 1a), and Kastoria (Figure 1b), Western Macedonia, Greece. The first was in September 2021, and the second in September 2022. The number of samples was one hundred and twenty (120) from Kastoria and one hundred and sixty (160) from Prespes for each sampling period, respectively. Samples came from various producers that followed traditional cultivation practices. After sampling, samples were kept frozen at −18 °C for 7 days so that all living components that could possibly ruin our samples were removed.

### 2.2. Sample Treatment

**Figure 2:**
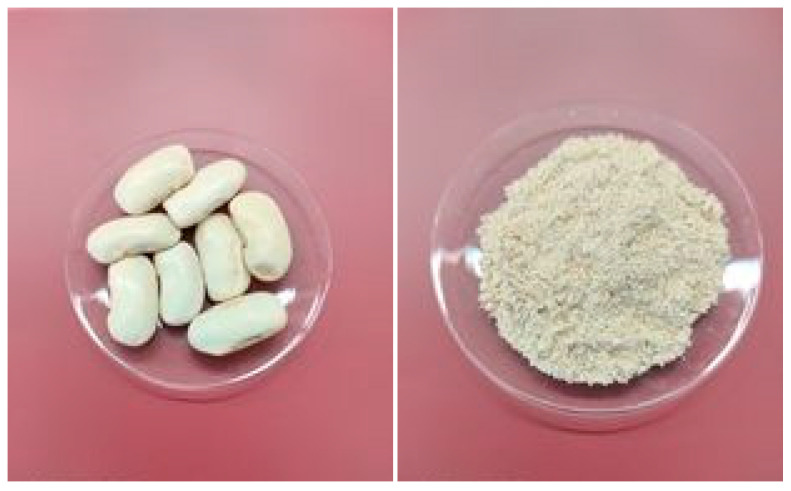
Giant beans before and after pulverization.

The first step for giant bean sample treatment was oven drying at 90 °C for 68 h. The next step was the grinding of the samples in a mill (pulverisette 11, Fritsch GmbH 93 Milling and Sizing, Idar-Oberstein, Germany) to fine powder, and then, they were stored in falcon tubes and placed in glass desiccators until IRMS analysis. Finally, bean samples were again oven-dried at 90 °C for 48 h and analyzed (Figure 2). The same sample treatment was followed in a previous study [24] that determined the nitrogen isotopic composition (δ15N) in common bean samples (*Phaseolus vulgaris*).

### 2.3. EA-IRMS Analysis

Aliquots of 5 mg were placed in tin capsules (4 × 4 × 11 mm, Elementar Analysensysteme GmbH, Hanau, Germany) and analyzed for carbon, nitrogen, and sulfur isotope ratios, e.g., ^13^C/^12^C, ^15^N/^14^N, and ^34^S/^32^S, using a continuous flow system consisting of an Elemental Analyser (Elementar Vario Isotope EL Cube, Elementar Analysensysteme GmbH, Hanau, Germany) coupled with an Elementar Isoprime 100 Isotope-Ratio Mass Spectrometry (IRMS) instrument (IsoPrime Ltd., Cheadle Hulme, UK). In the elemental analyzer, each sample was combusted quantitatively with oxygen added to the helium stream at a temperature of 1150 °C, and then NO_x_ and SO_x_ gases were reduced at 850 °C to produce N_2_ and SO_2_. The gases obtained were then introduced into the isotope ratio mass spectrometer.

The results of the isotope ratio analyses were expressed in permille (‰) using the delta δ notation and calculated according to the following equation:

*δ* Χ (‰) = [(R_sample_/R_standard_) − 1] × 1000

where X is the isotope being studied (e.g., ^13^C, ^15^N, ^34^S), R_sample_ is the isotopic ratio of the measured element in its physical form (e.g., ^13^C/^12^C, ^15^N/^14^N, and ^34^S/^32^S) in the sample, and R_standard_ is the isotopic ratio of the reference material. Before the bean samples analysis, a calibration of the IRMS instrument was carried out using reference materials of known compositions (standard substances). Each standard substance was selected according to the isotope that would be determined (C, N, or S). Moreover, a significant parameter for the selection of each reference material was its physicochemical properties, i.e., having isotope ratios close to the ones of the examined samples. The results were normalized to VPDB using ΙAΕA-600 (Caffeine, IAEA, Vienna, Austria), with assigned carbon isotope delta values and standard uncertainties (δ^13^C_V-PDB_ = −27.77‰ ± 0.043‰), to the atmospheric nitrogen (AIR-N_2_) using Β2155 (Protein IRMS Standard, Elemental Microanalysis Ltd., UK), with assigned nitrogen isotope delta values and standard uncertainties (δ^15^N_Air_ = 5.83‰ ± 0.08‰), and to the VCDT scale using ΙAΕA-S1 (Silver Sulfide, IAEA, Vienna, Austria), with assigned sulfur isotope delta values and standard uncertainties (δ^34^S_V-CDT_ = −0.3‰ ± 0.03‰). The results were extracted using the vario ISOTOPE cube V4.0.7 and IonVantage 1.7.3.0 for IsoPrime software. Each reference material was measured in duplicate at the beginning, after every four sample measurements, and at the end of each daily group of analyses.

Care was taken to ensure that the measured aliquots of reference materials and samples yielded a similar amount of gas. During sample analysis, a quality-control-check sample with B2159 (Sorghum Flour IRMS Standard, Elemental Microanalysis Ltd., UK with δ^13^C_V-PDB_ = −13.78‰, δ^15^N_Air_ = 1.58‰ and δ^34^S_V-CDT_ = 10.11‰) was analyzed to test the results of our samples.

### 2.4. Statistical Analysis

Statistical analysis was performed in order to visualize the distribution of all data obtained from the IRMS analysis from the region of Prespes (Florina) compared to those from Kastoria. For this reason, boxplot diagrams and histograms were used [25]. Furthermore, the discrimination of bean samples into their origin areas was performed. As already mentioned, it is essential to discriminate against value-added products and verify their origin and authenticity, especially in this case where beans are coming from two very close geographical areas.

The are many classification techniques used in general food quality evaluation [26]. However, for this case with two possible outcomes, a binary classification technique is required; therefore, binomial logistic regression was applied to the data to identify if it is possible to discriminate between these two areas based on the measured isotope ratios. Logistic regression is a statistical method that can be used to predict the probability of an event occurring based on observed features or variables. It is useful in cases where we want to model the event probability for a categorical response variable with two outcomes. For example, one or zero, true or false, yes or no, and for this case, if the sample came from Prespers or Kastroria. Since the probability of the described event (sample from Prespes or sample from Kastoria) must lie between 0 and 1, it is unrealistic to model probabilities with linear regression techniques because the linear regression model allows the dependent variable to take values greater than 1 or less than 0. The logistic regression model is a type of generalized linear model that extends the linear regression model by linking the range of real numbers to the range 0–1 [27]. The predicted probabilities can then be used to classify the data based on probability thresholds.

One critical result for the logistic regression is the calculated odd ratio for each predictive variable (in this case, the measured isotope ratios). In practice, the odds ratio compares the odds of two events and facilitates understanding the effect of the used predictors. The interpretation of an odds ratio depends on whether the predictor is categorical or continuous. For categorical predictors, the odds ratio compares the odds of the event occurring at two different levels of the predictor. For continuous predictors, such as the isotope ratios, odds ratios that are greater than 1 indicate that the event is more likely to occur as the predictor increases. Odds ratios that are less than 1 indicate that the event is less likely to occur as the predictor increases. It must be noted that the odds ratio differs from the risk, and while the odds may appear to be high, the absolute risk may be low [28]. In many reports, the odds ratio is the most commonly reported effect size, and it tends to be incorrectly interpreted as relative risk [29].

In addition, the predictive power of the estimated model was calculated by presenting sensitivity and specificity. The quantities of sensitivity and specificity are essential when we want to choose a threshold probability to turn a probability model into a classification model. Sensitivity (i.e., true positive rate) is the proportion of successful predictions that are correctly classified as such, while specificity (i.e., true negative rate) is the proportion of unsuccessful predictions that are correctly classified as such. The goal is to maximize both these rates, but there is often a trade-off between them. This trade-off can be visualized using a Receiver Operating Characteristic (ROC) curve, which allows the selection of an optimal threshold that maximizes sensitivity and specificity. Finally, the accuracy of the model was examined and calculated as the proportion of correct predictions over total predictions.

All computations were performed with the statistical software Jamovi ver.2.3.21., R studio ver. 24.04, and SPSS ver. 25.

## 3. Results and Discussion

### Stable Isotope Results for Giant Beans

One hundred and twenty (120) different bean samples from Kastoria and one hundred and sixty (160) different samples from Prespes for two different cultivation periods (2021 and 2022) were analyzed. For each sample, two repetitions were performed. The mean value of *δ*^15^N_AIR_ (‰) was 1.88 (min value −1.49, max value 7.05) for the Prespes area and 1.59 for the Kastoria area (min value −0.954, max value 6.01). The mean value of *δ*^13^C_V-PDB_ (‰) was −25.5 (min value −27.0, max value −22.06) for the Prespes area and −25.9 for the Kastoria area (min value −29.9, max value −23.3). The mean value of *δ*^34^S_V-CDT_ (‰) was 4.78 (min value 1.12, max value 8.95) for the Prespes area and −0.174 for the Kastoria area (min value −8.14, max value 8.19). These descriptive results of the analyzed samples are presented in Table 1. Briefly, the only stable isotope that seemed roughly different between the examined areas was the *δ*^34^S_V-CDT_ (‰). The mean values of the other examined isotopes were almost identical.

**Figure 3:**
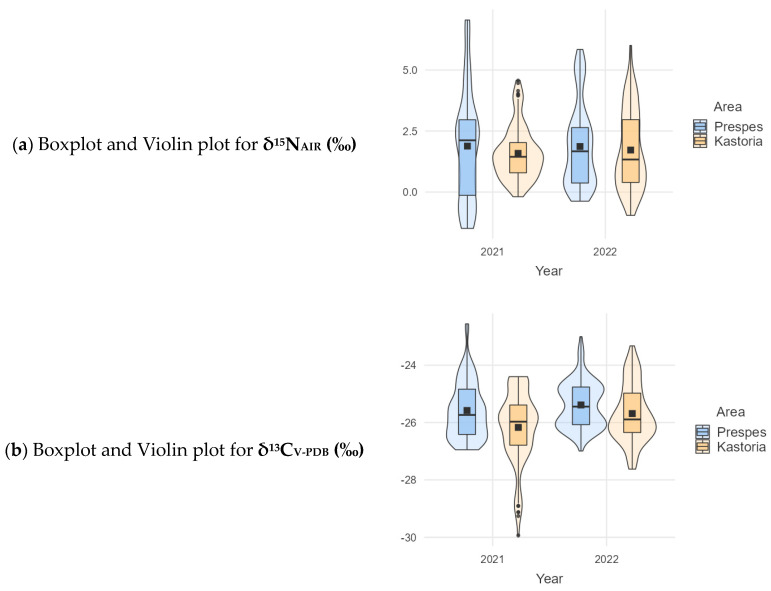

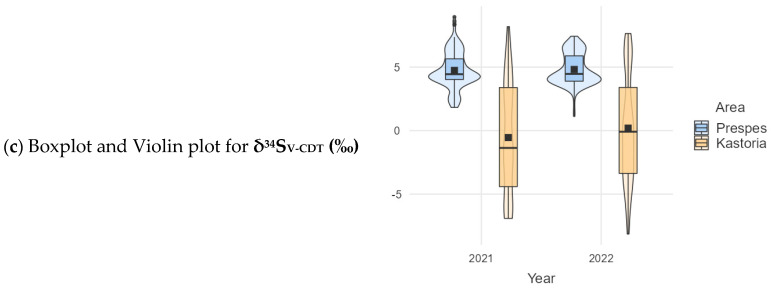
Boxplots and Violin plots of the analyzed isotopes.

Since the mean values were almost identical, it was sensible to examine the data distribution. Figure 3a–c presents the boxplots and violin plots for the measured δ values so that a better understanding of the data can be provided by visualizing the data and their distribution.

The next stage of the analysis was to run a one-way ANOVA for the mean values of *δ*^15^N_AIR_ (‰), *δ*^13^C_V-PDB_ (‰), and *δ*^34^S_V-CDT_ (‰) to find out if the examined areas and years presented statistically significant differences for their mean values. These results are presented in Table 2, Table 3 and Table 4.

The mean values of *δ*^13^C_V-PDB_ (‰) and *δ*^34^S_V-CDT_ (‰) were statistically different for the examined areas (Table 2 and Table 3, respectively). Even more, it seemed that the mean values of *δ*^15^N_AIR_ (‰) and *δ*^34^S_V-CDT_ (‰) were not affected by the different cultivation years (Table 4), while the mean values of *δ*^13^C_V-PDB_ (‰) were marginally statistically different for the examined years (Table 4). However, this result was based only on two different cultivation years, so it needs further examination.

Samples coming from different regions have different stable isotope ratios because of the different characteristics and environmental conditions, such as soil structure, climatic conditions, and soil nutrition. Therefore, stable isotope ratios were formulated due to this unique combination of characteristics [30]. Thus, this combination could affect the classification of products that have a specific value of the stable isotope ratio.

The next step of the analysis was to apply Binomial logistic regression to examine if it is possible to discriminate between these two close-neighbouring areas that cultivate beans using the measured values of *δ*^15^N_AIR_ (‰), *δ*^13^C_V-PDB_ (‰), and *δ*^34^S_V-CDT_ (‰) for years 2021 and 2022. In this direction, the binomial model proposed and examined used the examined area and the possibility of belonging in one of the two areas as the dependent variable, i.e., P(y = 1)/P(y = 0) that is the possibility of belonging to Prespes instead of Kastoria (P(Area = Prespes)/P(Area = Kastoria)). As the independent variables, the measured values of *δ* and a categorical variable named the Year, indicating the cultivating year (with the year 2021 used as a reference level), were used. Thus, the model underestimation is the following:

P(Area = Prespes)/P(Area = Kastoria) = constant/intercept + X*_δ_*^15^_NAIR (‰)_ + X*_δ_*^13^_CV-PDB (‰)_ + X*_δ_*^34^_SV-CDT (‰)_ + Year

Table 5 presents the results of the model estimation, that is, the coefficients of the independent variables along with pseudo-r-square statistics and the model’s overall goodness of fit. At this point, it should be noted that the r-square statistic cannot be exactly computed for logistic regression models, so the pseudo-r-square measures, named McFadden, Cox and Snell, and Nagelkerke approximations, were computed instead. In brief, larger pseudo-r-square statistics indicated that more of the variation was explained by the model to a maximum of 1, as was the case for linear regression. Finally, a goodness of fit test was performed, depicting that the estimated model adequately fits the data (see notes in Table 5).

As already mentioned, binary logistic regression is useful for situations in which a researcher wants to predict the presence or absence of a characteristic, or in this case, based on measured stable isotope rations to predict the area where the sample beans were cultivated, that is, Kastoria or Prespes. With that in mind, the predictive power of the estimated model should be examined. Table 6 presents the classification table of the estimated model.

Based on Table 6, it can be concluded that the estimated model could correctly classify bean samples to their area of origin using the values of *δ*^15^N_AIR_ (‰), *δ*^13^C_V-PDB_ (‰), and *δ*^34^S_V-CDT_ (‰). The accuracy of the model exceeded 78% for all samples. Having in mind that only three stable isotopes were examined in this study, it is safe to support that stable isotopes could discriminate close-neighboring areas producing the same products as the giant beans examined in this research. Nevertheless, samples from more cultivating periods are needed to determine how weather and climate affect the determination of the origin of an agricultural product. In a different approach different stable isotopes could be used to increase the accuracy of the prediction model.

Stable isotope ratio analysis was used for the determination of giant beans “Gigantes-Elefantes” PGI in two neighboring areas. Carbon and nitrogen isotopes provide information on plant or diet type. Also, nitrogen isotopes provide information about local agricultural practices, while sulfur isotope ratios depend on soil structure [31,32]. In a similar study, Opatic et al. (2016) [33] showed that carbon and sulfur isotopes can discriminate garlic and potato samples according to their geographical origin, even though they are grown in similar geological and climatic conditions. Likewise, Chung et al. (2019) [34] examined nitrogen and sulfur isotopes in dried shiitake slices and found that they varied significantly with country of origin. Park et al. (2019) [35] determined the ratios of carbon, nitrogen, and sulfur stable isotopes of onions produced in Korea and showed that this technique was highly efficient for the discrimination of Korean onions from those cultivated in other countries.

The results of the research indicate that more stable isotope ratios should be included in the analysis so that the accuracy of the prediction model could be increased. The next step is the analysis of other cultivation periods, as well as from other cultivation areas that produce “Gigantes-Elefantes” PGI beans and compare them with non-PGI samples. Nevertheless, this is the first study that makes available results that are useful for the discrimination of “Gigantes-Elefantes” PGI bean samples.

## 4. Conclusions

Stable isotope analysis is an analytical technique widely used for the discrimination of the geographical origin of many products. According to our knowledge, there are no previous studies conducted on stable isotope ratio analysis of ‘Gigantes-Elefantes” PGI beans. The purpose of this study was to investigate if the geographical origin of the ‘Gigantes-Elefantes” PGI beans could be differentiated according to the cultivation area in the same prefecture, Western Macedonia, Greece. In addition to stable isotope ratio analysis and the determination of δ^13^C, δ^15^N, and δ^34^S values, a one-way ANOVA and classification analysis were performed as the most common statistical approaches for the discrimination of the geographical origin. The results showed that carbon and sulfur δ‰ values were significantly different for the two sampling periods between the two areas. At this point, it should be mentioned that the two geographical areas considered in this work are relatively close to each other, and a significant difference in the measured δ^13^C values could appear unlikely to be plausible. For that reason, a different approach, named binomial logistic regression, was applied. This approach managed to improve the explanatory strength and correctly classify more than 78% of the samples. Based on the results of the three stable isotopes examined in our samples, sufficient discrimination can be achieved for products of different close origins and neighboring regions. However, more samples from different cultivation periods and the analysis of more stable isotopes, such as hydrogen and oxygen, could increase the accuracy of the prediction model.

## Figures and Tables

**Table 1:** Results of isotope analysis for the examined samples (mean δ values and s.d.).

Area	N (Samples)	Mean δ^15^N_AIR_ (‰)/ (S.D.)	Mean δ^13^C_V-PDB_ (‰)/ (S.D.)	Mean δ^34^S_V-CDT_ (‰)/ (S.D.)
2021				
Prespes	160	1.88 (2.26)	−25.6 (0.97)	4.74 (1.43)
Kastoria	120	1.59 (1.07)	−26.2 (1.23)	−0.54 (4.45)
2022				
Prespes	160	1.78 (1.68)	−25.4 (0.795)	4.82 (1.23)
Kastoria	120	1.72 (1.61)	−25.7 (0.985)	0.196 (4.21)

**Table 2:** One-way ANOVA analysis results for both years 2021 and 2022 (mean values and standard error).

*δ* (‰)	Area	N	Mean *δ* (‰)	Std. Error	ANOVA F Value	Sig.
δ^15^N_AIR_ (‰)	Prespes	320	1.875	0.111	2.44	0.119
	Kastoria	240	1.654	0.087		
δ^13^C_V-PDB_ (‰)	Prespes	320	−25.483	0.049	25.12	<0.001
	Kastoria	240	−25.928	0.073		
δ^34^S_V-CDT_ (‰)	Prespes	320	4.779	0.074	291.94	<0.001
	Kastoria	240	−0.174	0.280		

**Table 3:** One-way ANOVA analysis results for the Kastoria area for the years 2021 and 2022 (mean values and standard error).

*δ* (‰)	Years	N	Mean *δ* (‰)	Std. Error	ANOVA F Value	Sig.
δ^15^N_AIR_ (‰)	2021	120	1.587	0.097	0.588	0.444
	2022	120	1.722	0.146		
δ^13^C_V-PDB_ (‰)	2021	120	−26.169	0.112	11.244	<0.001
	2022	120	−25.687	0.089		
δ^34^S_V-CDT_ (‰)	2021	120	−0.545	0.406	1.757	0.186
	2022	120	0.196	0.384		

**Table 4:** One-way ANOVA analysis results for the Prespes area for the years 2021 and 2022 (mean values and standard error).

*δ* (‰)	Years	N	Mean *δ* (‰)	Std. Error	ANOVA F Value	Sig.
δ^15^N_AIR_ (‰)	2021	160	1.88	0.178	0.005	0.941
	2022	160	1.87	0.132		
δ^13^C_V-PDB_ (‰)	2021	160	−25.58	0.077	3.805	0.052
	2022	160	−25.39	0.062		
δ^34^S_V-CDT_ (‰)	2021	160	4.74	0.113	0.251	0.616
	2022	160	4.84	0.096		

**Table 5:** Binomial logistic regression to discriminate the cultivation area for the examined beans.

Predictor	Estimate	SE	Z	*p*	Odds Ratio
Intercept	21.562	3.9253	5.49	<0.001	2.31 × 10^9^
δ^15^N_AIR_ (‰)	−0.302	0.0693	−4.37	<0.001	0.739
δ^13^C_V-PDB_ (‰)	0.872	0.1522	5.73	<0.001	2.393
δ^34^S_V-CDT_ (‰)	0.626	0.0574	10.90	<0.001	1.871
Year:					
2022–2021	−0.467	0.2476	−1.89	0.059	0.627

Notes: Estimates represent the log odds of “Area = Prespes” vs. “Area = Kastoria”; Preudo R2 = 0.418 (McFadden), 0.435 (Cox & Snell), 0.854, (Nagelkerke); Overall goodness of Fit: X^2^ = 320, df = 4, *p* ≤ 0.001.

**Table 6:** Classification table of the examined binomial model.

Observed	Predicted		Percentage Correct
	Kastoria	Prespes	
Kastoria	188	52	78.3%
Prespes	70	250	78.1%

Notes: 1. The cut value is 0.665; 2. Accuracy = 0.782, Specificity = 0.783, Sensitivity = 0.781.

## Data Availability

The original contributions presented in the study are included in the article/Supplementary Materials, further inquiries can be directed to the corresponding author.

## Supplementary Materials

The following supporting information can be downloaded at: https://www.mdpi.com/article/10.3390/foods13132107/s1, Table S1: Sample data set from Prespes for the year 2021; Table S2: Sample data set from Prespes for the year 2022; Table S3: Sample data set from Kastoria for the year 2021; Table S4: Sample data set from Kastoria for the year 2022.
