# Supplementary material for: Stable Isotope Ratio Analysis for the Geographic Origin Discrimination of Greek Beans “Gigantes-Elefantes” (Phaseolus coccineus L.)
Source: Foods. 2024 Jul 2;13(13):2107. doi: 10.3390/foods13132107 (PMC11241270; doi:10.3390/foods13132107)
Supplement: Supplementary file 1 [file foods-13-02107-s001.zip › foods-3059131-supplementary.pdf]

Supplementary Material

Stable Isotope Ratio Analysis for the Geographic Origin Discrimination of Greek Beans "Gigantes-Elefantes" (*Phaseolus coccineus* L.)

Anna-Akrivi Thomatou, Eleni C. Mazarakioti, Anastasios Zotos, Efthimios Kokkotos, Achilleas Kontogeorgos, Angelos Patakas, Athanasios Ladavos

Table S1: Sample data set from Prespes for the year 2021

Sample number	Sample	$\delta^{15}N_{AIR}$ (‰)	$\delta^{13}C_{V\text{-}PDB}$ (‰)	$\delta^{34}S_{V\text{-}CDT}$ (‰)
1	Mik_GE_1	1.1773	-26.7357	4.0918
2	Mik_GE_2	2.7890	-24.0685	4.4423
3	Mik_GE_3	6.8248	-24.7234	4.5059
4	Mik_GE_4	1.2102	-26.4240	3.7544
5	Mik_GE_5	1.1494	-26.6359	3.6349
6	Mik_GE_6	1.1913	-26.8157	5.0608
7	Mik_GE_7	2.5669	-24.0626	4.4579
8	Mik_GE_8	3.1335	-24.0417	4.4437
9	Mik_GE_9	2.9451	-24.0529	4.4426
10	Mik_GE_10	6.9888	-23.6807	4.5601
11	Mik_GE_11	6.8439	-25.7667	4.5421
12	Mik_GE_12	6.8117	-24.8314	4.4392
13	Mik_GE_13	1.2004	-26.6435	3.5321
14	Mik_GE_14	0.9317	-26.6857	4.0625
15	Mik_GE_15	1.1287	-26.9106	4.6922
16	Mik_GE_16	2.6314	-24.1392	4.1327
17	Mik_GE_17	2.9641	-24.0449	4.4059
18	Mik_GE_18	2.8016	-24.0601	4.1363
19	Mik_GE_19	6.6469	-24.6786	4.5080
20	Mik_GE_20	6.6539	-24.7398	4.5427
21	Mik_GE_21	7.0488	-24.6870	4.4610
22	Mik_GE_22	1.1525	-26.6155	3.5077
23	Mik_GE_23	1.2432	-26.7037	4.0565
24	Mik_GE_24	1.2085	-26.6774	4.0289
25	Mik_GE_25	2.6022	-24.1186	4.6048
26	Mik_GE_26	3.0012	-24.1303	4.3908
27	Mik_GE_27	2.8161	-24.1974	4.4264
28	Mik_GE_28	6.9654	-24.8363	4.2519
29	Mik_GE_29	1.1653	-26.5810	3.7308
30	Mik_GE_30	1.1024	-26.5700	3.9123
31	Mik_GE_31	1.2086	-26.9485	4.7793

32	Mik_GE_32	2.5166	-24.1895	4.4583
33	Mik_GE_33	2.8293	-24.1366	4.4301
34	Mik_GE_34	2.9110	-24.0045	4.4392
35	Mik_GE_35	6.8161	-23.6803	4.3956
36	Mik_GE_36	6.6278	-24.7293	4.5461
37	Mik_GE_37	6.7616	-26.0495	4.5097
38	Mik_GE_38	1.2005	-26.5740	3.5155
39	Mik_GE_39	0.9988	-26.6935	4.0145
40	Mik_GE_40	1.2248	-26.8973	4.6773
41	Lekar_GE_1	4.8823	-24.5292	4.0273
42	Lekar_GE_2	2.4085	-25.8606	4.1725
43	Lekar_GE_3	2.9605	-24.9647	4.2355
44	Lekar_GE_4	-1.0446	-26.4436	4.1081
45	Lekar_GE_5	-0.2424	-25.5844	4.1788
46	Lekar_GE_6	3.4689	-25.3341	4.0898
47	Lekar_GE_7	5.0691	-22.6687	4.0598
48	Lekar_GE_8	4.5299	-24.4957	4.2934
49	Lekar_GE_9	4.9592	-24.4706	3.7664
50	Lekar_GE_10	5.0377	-24.6255	4.1168
51	Lekar_GE_11	2.1149	-25.7848	4.3149
52	Lekar_GE_12	2.6954	-25.9088	3.5519
53	Lekar_GE_13	2.4478	-25.8892	4.3373
54	Lekar_GE_14	2.6418	-24.5798	4.1221
55	Lekar_GE_15	3.1592	-24.4808	4.4886
56	Lekar_GE_16	3.0598	-25.1166	4.3003
57	Lekar_GE_17	-1.0427	-26.5810	4.1315
58	Lekar_GE_18	-1.0666	-26.2741	3.9522
59	Lekar_GE_19	-1.0679	-26.3250	4.1602
60	Lekar_GE_20	0.0305	-25.4505	4.2125
61	Lekar_GE_21	-0.3219	-25.3834	4.3689
62	Lekar_GE_22	-0.3916	-25.7057	3.9782
63	Lekar_GE_23	3.8706	-25.3028	3.9269
64	Lekar_GE_24	3.3651	-25.3154	4.1426
65	Lekar_GE_25	3.3111	-25.3643	3.9312
66	Lekar_GE_26	4.8502	-22.8155	4.2874
67	Lekar_GE_27	4.9665	-22.6294	4.1322
68	Lekar_GE_28	5.1674	-22.5566	4.1748
69	Lekar_GE_29	0.0420	-25.6089	4.2128
70	Lekar_GE_30	-0.3653	-25.5492	4.2174
71	Lekar_GE_31	-0.3550	-25.8494	3.9152
72	Lekar_GE_32	3.6501	-25.2286	3.9178
73	Lekar_GE_33	4.7154	-24.3769	4.3902
74	Lekar_GE_34	4.6203	-24.4694	4.0222
75	Lekar_GE_35	5.4504	-24.6594	3.6885
76	Lekar_GE_36	2.1114	-25.7626	4.5370
77	Lekar_GE_37	2.3880	-25.8846	3.7148

78	Lekar_GE_38	2.6492	-25.9296	4.2696
79	Lekar_GE_39	2.5931	-25.4772	3.8709
80	Lekar_GE_40	3.0188	-24.7805	4.6337
81	PL_GE_1	-0.0882	-25.1917	2.6063
82	PL_GE_2	2.8723	-25.4160	2.4547
83	PL_GE_3	-1.1974	-26.3746	2.2493
84	PL_GE_4	2.4345	-25.9841	7.0265
85	PL_GE_5	4.5523	-24.6724	5.7374
86	PL_GE_6	-1.2453	-26.7781	8.9483
87	PL_GE_7	0.5390	-25.8863	6.7298
88	PL_GE_8	0.1674	-25.6109	2.7006
89	PL_GE_9	-0.0823	-25.1387	2.5825
90	PL_GE_10	-0.0966	-25.2338	2.6978
91	PL_GE_11	-0.0769	-25.2851	2.4971
92	PL_GE_12	3.3076	-25.3126	2.2776
93	PL_GE_13	3.1762	-25.4055	2.6797
94	PL_GE_14	2.3318	-25.6689	2.2323
95	PL_GE_15	-0.9142	-26.1907	2.0199
96	PL_GE_16	-1.2400	-26.2651	1.8645
97	PL_GE_17	-1.4887	-26.5150	2.8150
98	PL_GE_18	2.5153	-25.9729	6.9662
99	PL_GE_19	2.6245	-26.0453	6.6714
100	PL_GE_20	2.3253	-26.1077	7.3681
101	PL_GE_21	4.4201	-24.6578	5.0683
102	PL_GE_22	4.5600	-24.5687	6.1912
103	PL_GE_23	4.6203	-24.7964	6.2754
104	PL_GE_24	-1.0770	-26.7702	8.6062
105	PL_GE_25	-1.2292	-26.8975	8.3418
106	PL_GE_26	-1.4372	-26.7189	8.3548
107	PL_GE_27	0.5686	-25.9276	6.6820
108	PL_GE_28	0.6491	-25.9184	6.9054
109	PL_GE_29	0.4322	-25.9259	6.9233
110	PL_GE_30	0.4731	-25.3485	2.4551
111	PL_GE_31	0.0618	-25.6635	2.4818
112	PL_GE_32	0.0143	-25.8239	2.8352
113	PL_GE_33	-1.0601	-26.2115	1.9768
114	PL_GE_34	-1.3001	-26.2681	2.0077
115	PL_GE_35	-1.4521	-26.2956	1.8247
116	PL_GE_36	-1.2053	-26.6783	2.7191
117	PL_GE_37	2.3915	-26.0650	6.8496
118	PL_GE_38	2.6405	-25.8018	7.0788
119	PL_GE_39	2.2503	-25.9323	7.1635
120	PL_GE_40	4.4103	-24.6651	4.9420
121	Pil_GE_1	2.3334	-26.7677	5.4047
122	Pil_GE_2	-1.0036	-26.5454	5.2429
123	Pil_GE_3	-0.5460	-26.2077	5.9838

124	Pil_GE_4	1.9308	-25.3522	6.4758
125	Pil_GE_5	2.3550	-25.4934	6.3523
126	Pil_GE_6	2.0265	-26.8250	5.5210
127	Pil_GE_7	2.2205	-26.7479	5.2233
128	Pil_GE_8	2.3906	-26.7795	5.4475
129	Pil_GE_9	-1.0266	-26.5466	5.1677
130	Pil_GE_10	-0.9813	-26.5479	5.3806
131	Pil_GE_11	-0.9260	-26.5540	5.0977
132	Pil_GE_12	-0.3696	-26.3055	6.2533
133	Pil_GE_13	-0.6625	-26.1213	5.9880
134	Pil_GE_14	-0.6456	-26.1091	5.7412
135	Pil_GE_15	2.2952	-25.3652	7.2028
136	Pil_GE_16	1.8801	-25.2596	6.2516
137	Pil_GE_17	1.7558	-25.3471	6.0069
138	Pil_GE_18	2.1268	-25.4301	6.7799
139	Pil_GE_19	2.6722	-25.4974	6.4352
140	Pil_GE_20	2.3261	-25.5623	6.2606
141	Pil_GE_21	2.0033	-26.8122	5.4772
142	Pil_GE_22	2.4274	-26.7238	5.2005
143	Pil_GE_23	2.5003	-26.7529	5.4471
144	Pil_GE_24	-1.0413	-26.5588	4.9485
145	Pil_GE_25	-0.8642	-26.5284	5.2907
146	Pil_GE_26	-0.8823	-26.5430	5.3229
147	Pil_GE_27	-0.3860	-26.2520	6.4619
148	Pil_GE_28	-0.6329	-26.2495	5.9867
149	Pil_GE_29	-0.6440	-26.1235	5.5496
150	Pil_GE_30	2.1404	-25.3400	6.9446
151	Pil_GE_31	-0.9717	-26.5405	5.4607
152	Pil_GE_32	-0.9975	-26.5581	5.1706
153	Pil_GE_33	-0.3532	-26.4109	6.4334
154	Pil_GE_34	-0.6178	-26.1891	5.9323
155	Pil_GE_35	-0.6347	-26.0469	5.6238
156	Pil_GE_36	2.4103	-25.3592	6.4843
157	Pil_GE_37	1.7506	-25.3710	6.7384
158	Pil_GE_38	1.9757	-25.3932	6.2646
159	Pil_GE_39	2.1148	-25.3885	5.9681
160	Pil_GE_40	2.4546	-25.4752	6.4402

Table S2: Sample data set from Prespes for the year 2022

Sample number	Sample	$\delta^{15}N_{AIR}$ (‰)	$\delta^{13}C_{V-PDB}$ (‰)	$\delta^{34}S_{V-CDT}$ (‰)
1	Mik_GE_1/R2	3.2820	-24.9940	4.6807
2	Mik_GE_2/R2	2.4063	-24.4583	4.6430
3	Mik_GE_3/R2	2.9277	-24.9617	4.6663
4	Mik_GE_4/R2	2.1963	-24.6150	3.6173
5	Mik_GE_5/R2	3.2110	-24.7887	4.2127
6	Mik_GE_6/R2	2.2360	-24.8893	4.3600
7	Mik_GE_7/R2	3.1367	-25.0693	3.7237
8	Mik_GE_8/R2	2.4837	-24.7060	4.2173
9	Mik_GE_9/R2	1.6957	-24.9053	4.1790
10	Mik_GE_10/R2	3.5213	-25.2200	3.8873
11	Mik_GE_11/R2	2.2637	-25.0380	3.9603
12	Mik_GE_12/R2	1.2490	-24.6663	4.1103
13	Mik_GE_13/R2	1.7700	-24.5613	3.7913
14	Mik_GE_14/R2	1.4667	-24.9001	3.9096
15	Mik_GE_15/R2	4.0255	-24.3687	4.0882
16	Mik_GE_16/R2	2.5916	-24.7044	3.8560
17	Mik_GE_17/R2	4.4334	-24.6857	3.9370
18	Mik_GE_18/R2	4.6569	-23.6517	3.5718
19	Mik_GE_19/R2	2.8016	-24.6044	4.5227
20	Mik_GE_20/R2	4.6165	-24.5747	4.6823
21	Mik_GE_21/R2	2.9899	-24.6617	3.8553
22	Mik_GE_22/R2	2.9762	-24.3066	3.7569
23	Mik_GE_23/R2	2.9831	-24.4716	3.8427
24	Mik_GE_24/R2	2.4743	-24.8847	3.7477
25	Mik_GE_25/R2	2.4603	-24.2843	4.2645
26	Mik_GE_26/R2	3.0615	-24.8368	4.3243
27	Mik_GE_27/R2	1.9434	-24.6821	4.0935
28	Mik_GE_28/R2	3.1202	-23.2767	3.7414
29	Mik_GE_29/R2	2.6124	-23.4713	3.9827
30	Mik_GE_30/R2	2.0874	-23.6627	3.8622
31	Mik_GE_31/R2	2.3775	-23.5126	3.8941
32	Mik_GE_32/R2	2.1272	-24.9920	4.1091
33	Mik_GE_33/R2	2.3818	-24.6791	4.4043
34	Mik_GE_34/R2	2.8044	-23.9829	4.1968
35	Mik_GE_35/R2	2.4366	-24.7027	3.3388
36	Mik_GE_36/R2	2.0915	-24.5350	3.9207
37	Mik_GE_37/R2	1.9543	-24.6269	3.5109
38	Mik_GE_38/R2	2.5752	-23.0038	4.5912
39	Mik_GE_39/R2	2.9383	-24.7609	3.7080
40	Mik_GE_40/R2	3.6996	-23.6123	4.5874
41	Lekar_GE_1/R2	1.7528	-26.1399	4.1378
42	Lekar_GE_2/R2	1.4597	-26.5683	4.1809
43	Lekar_GE_3/R2	1.8114	-26.3520	4.5945
44	Lekar_GE_4/R2	1.4922	-25.2067	3.5891

45	Lekar_GE_5/R2	1.7992	-26.2064	4.6013
46	Lekar_GE_6/R2	1.7215	-25.9009	4.2037
47	Lekar_GE_7/R2	1.2339	-25.1484	3.7217
48	Lekar_GE_8/R2	1.2169	-25.6895	4.5798
49	Lekar_GE_9/R2	1.8742	-26.0192	4.5118
50	Lekar_GE_10/R2	0.8855	-25.7870	4.0893
51	Lekar_GE_11/R2	1.1232	-25.8280	3.8405
52	Lekar_GE_12/R2	2.3142	-25.5560	4.3093
53	Lekar_GE_13/R2	1.6453	-26.2953	3.7215
54	Lekar_GE_14/R2	1.6109	-26.4512	3.8080
55	Lekar_GE_15/R2	0.9752	-26.3405	4.5544
56	Lekar_GE_16/R2	1.7144	-25.7320	4.7088
57	Lekar_GE_17/R2	2.4219	-25.3759	3.8724
58	Lekar_GE_18/R2	2.0173	-24.5711	3.3715
59	Lekar_GE_19/R2	2.3290	-24.9674	1.4797
60	Lekar_GE_20/R2	1.8971	-25.7984	3.7932
61	Lekar_GE_21/R2	0.0517	-25.0497	3.9149
62	Lekar_GE_22/R2	0.0973	-24.3836	3.7815
63	Lekar_GE_23/R2	0.0840	-24.5378	3.4631
64	Lekar_GE_24/R2	0.6306	-24.7050	4.2455
65	Lekar_GE_25/R2	0.8388	-24.2055	3.4967
66	Lekar_GE_26/R2	0.1815	-24.7580	4.0335
67	Lekar_GE_27/R2	0.3732	-24.6741	3.2889
68	Lekar_GE_28/R2	-0.0844	-24.2326	5.3899
69	Lekar_GE_29/R2	0.3272	-24.8361	4.1442
70	Lekar_GE_30/R2	0.3478	-24.4644	3.7544
71	Lekar_GE_31/R2	0.5891	-25.5387	3.6708
72	Lekar_GE_32/R2	0.7274	-24.8528	3.7892
73	Lekar_GE_33/R2	0.2631	-24.9551	4.0505
74	Lekar_GE_34/R2	0.5541	-24.5387	3.8724
75	Lekar_GE_35/R2	1.0434	-25.1374	3.3792
76	Lekar_GE_36/R2	0.8729	-25.9741	4.0470
77	Lekar_GE_37/R2	0.2546	-24.8019	2.9154
78	Lekar_GE_38/R2	0.3416	-25.7672	3.3352
79	Lekar_GE_39/R2	1.1352	-24.6914	3.7944
80	Lekar_GE_40/R2	0.9493	-25.5680	4.9072
81	PL_GE_1/R2	5.0506	-25.3680	3.9058
82	PL_GE_2/R2	4.9681	-25.2464	4.4696
83	PL_GE_3/R2	4.7476	-25.1775	4.2180
84	PL_GE_4/R2	4.9701	-25.4446	4.4598
85	PL_GE_5/R2	4.8352	-25.9304	4.1415
86	PL_GE_6/R2	5.1701	-25.1712	4.2907
87	PL_GE_7/R2	4.9114	-26.0164	4.0774
88	PL_GE_8/R2	5.0639	-24.8806	4.2184
89	PL_GE_9/R2	5.0439	-24.7421	4.0419
90	PL_GE_10/R2	5.1232	-25.2767	5.4126

91	PL_GE_11/R2	5.2708	-26.0822	4.3331
92	PL_GE_12/R2	5.2225	-25.0323	4.5979
93	PL_GE_13/R2	5.2181	-26.3937	4.6134
94	PL_GE_14/R2	5.5622	-25.1721	4.6703
95	PL_GE_15/R2	5.6102	-25.6858	4.4127
96	PL_GE_16/R2	5.1089	-26.3390	3.6375
97	PL_GE_17/R2	5.3515	-24.9719	4.3065
98	PL_GE_18/R2	5.4181	-24.7590	3.7883
99	PL_GE_19/R2	5.8417	-25.6447	4.6668
100	PL_GE_20/R2	5.3218	-25.2346	5.3637
101	PL_GE_21/R2	1.9833	-24.9771	5.6844
102	PL_GE_22/R2	2.6048	-24.9508	5.5631
103	PL_GE_23/R2	2.8532	-25.7030	5.3753
104	PL_GE_24/R2	2.5088	-25.6607	6.0826
105	PL_GE_25/R2	2.6689	-25.4425	4.9898
106	PL_GE_26/R2	2.1828	-25.0434	5.2708
107	PL_GE_27/R2	2.4624	-25.2463	5.3016
108	PL_GE_28/R2	2.2860	-25.2642	5.4095
109	PL_GE_29/R2	2.6316	-25.5056	4.9718
110	PL_GE_30/R2	2.3758	-25.6999	2.2025
111	PL_GE_31/R2	1.2025	-26.1533	6.0067
112	PL_GE_32/R2	0.2319	-26.8953	6.4571
113	PL_GE_33/R2	0.3571	-25.7094	5.3826
114	PL_GE_34/R2	1.4788	-26.2505	6.7981
115	PL_GE_35/R2	1.1461	-26.3986	6.4849
116	PL_GE_36/R2	0.8504	-26.1328	6.3565
117	PL_GE_37/R2	1.7821	-25.8212	7.1685
118	PL_GE_38/R2	0.9312	-26.2771	6.9657
119	PL_GE_39/R2	1.2858	-26.9920	6.5253
120	PL_GE_40/R2	1.1867	-25.2435	1.1169
121	Pil_GE_1/R2	0.2287	-26.4513	5.9809
122	Pil_GE_2/R2	0.0301	-26.1269	6.0402
123	Pil_GE_3/R2	0.7362	-26.0541	6.4569
124	Pil_GE_4/R2	0.5577	-26.1563	5.4902
125	Pil_GE_5/R2	0.2710	-25.8690	6.1890
126	Pil_GE_6/R2	0.3671	-25.8058	5.9206
127	Pil_GE_7/R2	0.4602	-26.5035	5.9729
128	Pil_GE_8/R2	0.5088	-25.6490	5.2288
129	Pil_GE_9/R2	0.2781	-25.9283	6.2363
130	Pil_GE_10/R2	0.5845	-26.2484	6.1127
131	Pil_GE_11/R2	0.0565	-26.2270	5.8763
132	Pil_GE_12/R2	0.5560	-26.2272	6.1860
133	Pil_GE_13/R2	0.5041	-26.0707	5.4634
134	Pil_GE_14/R2	0.3719	-25.8992	5.6769
135	Pil_GE_15/R2	0.5514	-26.5589	5.8094
136	Pil_GE_16/R2	0.1283	-25.9900	5.3952

137	Pil_GE_17/R2	0.0605	-26.4746	5.7923
138	Pil_GE_18/R2	0.3333	-25.8166	5.2580
139	Pil_GE_19/R2	0.1252	-26.1570	6.0998
140	Pil_GE_20/R2	0.2581	-26.1553	6.7205
141	Pil_GE_21/R2	0.0737	-26.2536	6.4633
142	Pil_GE_22/R2	0.1403	-25.8989	6.6603
143	Pil_GE_23/R2	0.2934	-26.4796	6.5070
144	Pil_GE_24/R2	0.3167	-26.2567	6.5716
145	Pil_GE_25/R2	0.0326	-26.2011	7.1742
146	Pil_GE_26/R2	0.4161	-26.2869	6.5244
147	Pil_GE_27/R2	-0.1960	-25.8598	6.5748
148	Pil_GE_28/R2	0.0525	-26.1877	6.8838
149	Pil_GE_29/R2	-0.0087	-26.3710	6.7056
150	Pil_GE_30/R2	-0.3552	-25.9725	6.8374
151	Pil_GE_31/R2	-0.0783	-26.2211	7.4320
152	Pil_GE_32/R2	0.2194	-25.8586	6.4612
153	Pil_GE_33/R2	-0.0890	-25.9820	6.5815
154	Pil_GE_34/R2	0.5418	-25.9824	7.0804
155	Pil_GE_35/R2	-0.3729	-26.2355	6.9133
156	Pil_GE_36/R2	0.3787	-25.9149	6.9128
157	Pil_GE_37/R2	-0.1924	-26.1351	6.8117
158	Pil_GE_38/R2	0.2639	-25.9633	6.8310
159	Pil_GE_39/R2	-0.2584	-26.1990	6.9082
160	Pil_GE_40/R2	-0.1435	-26.2487	6.8246

Table S3: Sample data set from Kastoria for the year 2021

Sample number	Sample	$\delta^{15}N_{AIR}$ (‰)	$\delta^{13}C_{V\text{-}PDB}$ (‰)	$\delta^{34}S_{V\text{-}CDT}$ (‰)
1	Pol_1	4.1486	-25.9721	5.1573
2	Pol_2	0.7467	-24.4574	5.4864
3	Pol_3	3.9488	-25.9915	6.0948
4	Pol_4	3.8184	-25.9758	6.1687
5	Pol_5	4.5717	-25.9544	5.0640
6	Pol_6	0.8182	-24.4264	5.4259
7	Pol_7	0.7186	-24.4675	5.1900
8	Pol_8	0.7436	-24.4909	5.3573
9	Pol_9	4.0207	-25.7556	5.4131
10	Pol_10	4.0061	-25.9506	4.9181
11	Pol_11	4.4625	-25.9302	5.1277
12	Pol_12	0.8257	-24.4343	5.0695
13	Pol_13	0.7398	-24.4566	5.8722
14	Pol_14	0.6128	-24.4992	5.3229
15	Pol_15	3.9618	-25.9660	5.1692
16	Lit_1	0.4252	-25.5735	7.4787
17	Lit_2	2.7962	-26.8111	7.9306
18	Lit_3	2.3331	-27.1432	3.4589
19	Lit_4	1.5810	-29.1242	3.6738
20	Lit_5	1.1589	-27.7782	3.8161
21	Lit_6	0.1125	-27.0215	3.7772
22	Lit_7	2.3934	-24.4568	3.7071
23	Lit_8	2.8205	-26.6766	7.2954
24	Lit_9	1.9405	-26.6638	7.4639
25	Lit_10	3.0889	-27.1243	8.1893
26	Lit_11	2.3583	-26.6881	3.5064
27	Lit_12	2.5227	-27.6889	3.3784
28	Lit_13	2.6951	-27.0014	3.4728
29	Lit_14	1.6593	-28.8272	3.9926
30	Lit_15	1.4360	-29.2622	3.4285
31	AO_1	1.5530	-26.0606	-3.2689
32	AO_2	1.8411	-28.8153	-3.3472
33	AO_3	1.1636	-28.9104	-3.7106
34	AO_4	1.5119	-25.4370	2.7905
35	AO_5	3.6658	-25.2819	2.5402
36	AO_6	1.4223	-25.9103	2.9306
37	AO_7	2.5512	-26.0997	2.2160
38	AO_8	2.5674	-26.7181	1.2229
39	AO_9	0.6070	-26.4356	1.1409
40	AO_10	1.1268	-26.6678	-1.3870
41	AO_11	0.9874	-25.0705	-1.6204
42	AO_12	1.5206	-26.1163	-3.6397
43	AO_13	1.3643	-26.2345	-2.9259

44	AO_14	1.6246	-28.5521	-3.2381
45	AO_15	1.6712	-28.9060	-3.1605
46	AO_16	2.1395	-29.1273	-3.6485
47	AO_17	1.2582	-28.4714	-3.4196
48	AO_18	1.2125	-28.5226	-4.0820
49	AO_19	1.1976	-29.9352	-3.7490
50	AO_20	1.0339	-25.4059	2.5990
51	AO_21	1.6577	-25.3203	2.3782
52	AO_22	1.7645	-25.4145	2.9404
53	AO_23	3.6156	-25.3109	2.2880
54	AO_24	3.5717	-25.1411	3.1906
55	AO_25	3.1297	-25.3273	2.3513
56	AO_26	1.4536	-25.6045	3.4370
57	AO_27	1.4065	-25.7311	2.9252
58	AO_28	1.4917	-26.1132	2.9538
59	AO_29	2.4743	-25.7406	2.3692
60	AO_30	2.5399	-26.0126	2.8047
61	AO_31	2.5362	-26.0041	2.7898
62	AO_32	2.2531	-26.9431	1.0925
63	AO_33	2.8644	-26.7828	-0.0178
64	AO_34	2.6103	-26.4066	1.0057
65	AO_35	0.7215	-26.3600	0.7547
66	Kal_1	0.3782	-24.5828	-5.8898
67	Kal_2	0.0417	-25.7051	-6.5537
68	Kal_3	0.9836	-25.8023	-6.4934
69	Kal_4	1.2651	-24.6461	-6.4201
70	Kal_5	0.9738	-25.1965	-6.2123
71	Kal_6	0.3121	-24.4899	-5.9342
72	Kal_7	0.3822	-24.5982	-6.9134
73	Kal_8	0.4380	-24.6751	-6.3855
74	Kal_9	-0.0163	-25.7574	-6.3303
75	Kal_10	0.1080	-25.6546	-6.2955
76	Kal_11	-0.1913	-25.7061	-6.1357
77	Kal_12	0.9645	-25.7811	-6.6406
78	Kal_13	0.7361	-25.6695	-6.6194
79	Kal_14	1.1321	-26.0260	-5.7855
80	Kal_15	1.2811	-24.4326	-6.1755
81	Kal_16	1.3498	-24.5788	-5.3917
82	Kal_17	1.2224	-24.8413	-6.5202
83	Kal_18	1.1479	-24.9865	-6.6981
84	Kal_19	0.9555	-25.1930	-6.3338
85	Kal_20	0.8598	-25.3322	-6.5449
86	Kal_21	0.4497	-24.5055	-6.1626
87	Kal_22	0.2174	-24.4425	-5.9311
88	Kal_23	0.4697	-24.7062	-6.2063
89	Kal_24	0.1678	-25.7847	-6.5566

90	Kal_25	0.2158	-25.6810	-6.6502
91	Kal_26	0.0705	-25.6737	-6.6570
92	Kal_27	1.0831	-25.7564	-6.8335
93	Kal_28	0.8035	-25.7686	-5.7173
94	Kal_29	1.0805	-25.8412	-6.3218
95	Kal_30	1.1196	-24.3987	-6.6137
96	Lak_1	1.7006	-26.9301	-1.3676
97	Lak_2	0.5616	-27.1777	-1.2662
98	Lak_3	1.5291	-26.6093	-1.5591
99	Lak_4	1.9755	-26.4235	-1.6620
100	Lak_5	1.8262	-25.6974	-1.5480
101	Lak_6	1.9498	-26.6159	-1.3939
102	Lak_7	1.5611	-26.9904	-0.9945
103	Lak_8	1.7406	-27.0632	-1.1418
104	Lak_9	0.5632	-27.2759	-1.4421
105	Lak_10	0.5381	-27.2200	-2.3712
106	Lak_11	0.6641	-27.0024	-1.9310
107	Lak_12	1.8262	-26.5881	-1.4996
108	Lak_13	1.7450	-26.6440	-1.1969
109	Lak_14	1.2633	-26.5764	-1.0023
110	Lak_15	2.1094	-26.3811	-1.3566
111	Lak_16	1.7719	-26.2808	-1.2497
112	Lak_17	1.9977	-26.5584	-1.5566
113	Lak_18	1.7080	-25.5736	-1.6749
114	Lak_19	1.9130	-25.6640	-1.2951
115	Lak_20	1.9042	-25.8401	-1.9105
116	Lak_21	1.8039	-26.7435	-2.1427
117	Lak_22	1.5242	-26.9434	-1.6464
118	Lak_23	1.7669	-27.1256	-1.9610
119	Lak_24	0.4199	-27.4323	-1.5618
120	Lak_25	0.4881	-27.2240	-1.6861

Table S4: Sample data set from Kastoria for the year 2022

Sample number	Sample	$\delta^{15}N_{AIR}$ (‰)	$\delta^{13}C_{V\text{-}PDB}$ (‰)	$\delta^{34}S_{V\text{-}CDT}$ (‰)
1	Pol_1/R2	4.0253	-26.5255	6.6413
2	Pol_2/R2	1.7891	-26.6291	6.5260
3	Pol_3/R2	2.7922	-26.8318	6.4197
4	Pol_4/R2	2.4881	-26.2797	6.6055
5	Pol_5/R2	4.1513	-26.3290	6.6006
6	Pol_6/R2	4.5507	-26.3574	6.5826
7	Pol_7/R2	4.2784	-27.2140	7.1649
8	Pol_8/R2	5.7726	-26.6258	6.6209
9	Pol_9/R2	3.9779	-26.3090	6.6861
10	Pol_10/R2	2.0369	-26.2418	6.6731
11	Pol_11/R2	4.6918	-25.5918	6.5022
12	Pol_12/R2	6.0085	-26.4963	6.3514
13	Pol_13/R2	3.4706	-26.6797	6.8007
14	Pol_14/R2	4.4703	-26.6490	6.7774
15	Pol_15/R2	3.8491	-26.5794	6.4778
16	Lit_1/R2	3.2083	-24.8390	7.6485
17	Lit_2/R2	3.6957	-25.0384	4.6805
18	Lit_3/R2	2.7180	-25.5717	5.3550
19	Lit_4/R2	2.9810	-25.7994	5.5066
20	Lit_5/R2	3.1254	-24.9738	5.7293
21	Lit_6/R2	3.0213	-25.1896	6.0344
22	Lit_7/R2	3.0564	-24.3330	6.2350
23	Lit_8/R2	3.7796	-25.9302	6.0984
24	Lit_9/R2	2.9539	-26.2271	5.9440
25	Lit_10/R2	1.3138	-24.8552	5.7395
26	Lit_11/R2	1.4703	-25.8622	5.2009
27	Lit_12/R2	1.2152	-24.7626	6.1070
28	Lit_13/R2	1.0156	-25.9642	4.8513
29	Lit_14/R2	0.7229	-24.3708	5.9155
30	Lit_15/R2	1.2815	-25.4425	6.3837
31	AO_1/R2	0.0889	-27.5106	-6.2551
32	AO_2/R2	-0.4056	-27.6249	-8.1434
33	AO_3/R2	-0.3775	-27.4949	-6.9761
34	AO_4/R2	0.1965	-26.6507	-6.6098
35	AO_5/R2	0.7070	-27.4007	-5.6553
36	AO_6/R2	-0.9536	-27.2505	-6.7772
37	AO_7/R2	0.0911	-27.3363	-6.7665
38	AO_8/R2	0.7354	-27.2607	-7.5274
39	AO_9/R2	0.5809	-27.1716	-6.3319
40	AO_10/R2	1.6544	-26.3946	0.2647
41	AO_11/R2	1.1958	-26.1609	0.1071
42	AO_12/R2	1.6272	-25.9021	0.5129
43	AO_13/R2	1.1861	-26.5600	0.1710

44	AO_14/R2	1.6422	-26.3083	0.2396
45	AO_15/R2	1.3535	-25.6443	0.8280
46	AO_16/R2	1.0849	-26.2508	0.0841
47	AO_17/R2	1.3610	-25.6984	1.0622
48	AO_18/R2	1.1364	-26.4925	0.9237
49	AO_19/R2	1.3666	-25.6353	2.5054
50	AO_20/R2	0.7833	-25.5221	2.5921
51	AO_21/R2	1.5953	-25.4188	2.5027
52	AO_22/R2	1.5652	-25.2229	2.7459
53	AO_23/R2	2.4614	-25.2203	1.8056
54	AO_24/R2	1.8176	-25.5272	2.0714
55	AO_25/R2	1.2798	-25.5174	2.7039
56	AO_26/R2	1.0628	-24.6769	2.4577
57	AO_27/R2	1.2559	-25.8770	2.9755
58	AO_28/R2	2.2898	-25.8952	-0.5094
59	AO_29/R2	2.5999	-26.3316	-1.2969
60	AO_30/R2	2.2218	-25.5659	-0.4001
61	AO_31/R2	2.6064	-25.8910	1.2078
62	AO_32/R2	2.2117	-25.9870	-1.3111
63	AO_33/R2	2.2792	-25.8179	-0.0641
64	AO_34/R2	2.6837	-26.1915	-0.3910
65	AO_35/R2	2.8393	-26.3946	-1.7104
66	Kal_1/R2	0.5520	-24.1040	-1.8439
67	Kal_2/R2	0.6581	-23.9875	-1.5887
68	Kal_3/R2	0.6275	-23.3246	-2.2344
69	Kal_4/R2	0.8914	-24.0600	-2.0840
70	Kal_5/R2	0.2922	-24.1160	-1.9928
71	Kal_6/R2	1.4160	-23.9112	-2.1150
72	Kal_7/R2	0.9116	-24.5503	-1.9300
73	Kal_8/R2	0.6133	-23.7115	-1.4590
74	Kal_9/R2	0.9364	-24.1933	-1.8480
75	Kal_10/R2	-0.0832	-25.8905	-3.9059
76	Kal_11/R2	0.1938	-26.7033	-4.2279
77	Kal_12/R2	-0.0604	-25.9595	-3.7472
78	Kal_13/R2	0.3772	-26.0038	-4.4436
79	Kal_14/R2	0.8025	-25.7770	-3.8639
80	Kal_15/R2	0.3384	-26.1780	-3.7786
81	Kal_16/R2	0.0165	-25.8707	-4.4664
82	Kal_17/R2	-0.1319	-26.4547	-4.1071
83	Kal_18/R2	-0.8346	-26.1112	-4.0346
84	Kal_19/R2	-0.1041	-26.3437	-4.0040
85	Kal_20/R2	-0.0864	-25.7521	-3.9541
86	Kal_21/R2	-0.0409	-26.4295	-4.7145
87	Kal_22/R2	0.3497	-26.1350	-3.4868
88	Kal_23/R2	0.6542	-26.1348	-4.3137
89	Kal_24/R2	-0.2896	-26.1553	-3.7901

90	Kal_25/R2	-0.0234	-26.0226	-3.8967
91	Kal_26/R2	0.5179	-26.3840	-4.4071
92	Kal_27/R2	0.4961	-25.7178	-3.9247
93	Kal_28/R2	1.0459	-24.0712	-2.6720
94	Kal_29/R2	0.1482	-23.7898	-2.4971
95	Kal_30/R2	0.9529	-23.4142	-1.6526
96	Lak_1/R2	4.1915	-24.9712	0.1972
97	Lak_2/R2	4.0489	-24.6856	-0.5464
98	Lak_3/R2	4.0420	-24.2967	-0.4920
99	Lak_4/R2	3.9235	-24.2843	0.1146
100	Lak_5/R2	3.7627	-24.0832	-0.3088
101	Lak_6/R2	4.7677	-24.1935	0.1865
102	Lak_7/R2	4.1202	-23.7316	-0.1106
103	Lak_8/R2	3.4329	-25.5032	-0.2180
104	Lak_9/R2	4.5463	-24.7992	-0.3974
105	Lak_10/R2	-0.2316	-25.9780	-3.3319
106	Lak_11/R2	0.2704	-26.3409	-3.2736
107	Lak_12/R2	0.0725	-26.4630	-3.1173
108	Lak_13/R2	0.4016	-26.0422	-3.3532
109	Lak_14/R2	-0.3667	-26.6569	-3.7838
110	Lak_15/R2	-0.7500	-26.4280	-3.4173
111	Lak_16/R2	-0.1904	-26.3386	-3.4402
112	Lak_17/R2	-0.6528	-25.4670	-3.1350
113	Lak_18/R2	0.1004	-26.3013	-3.2341
114	Lak_19/R2	3.0108	-24.9411	1.1866
115	Lak_20/R2	3.4956	-24.8419	0.3861
116	Lak_21/R2	2.0355	-24.1025	0.4341
117	Lak_22/R2	2.8332	-24.6648	0.8136
118	Lak_23/R2	2.5860	-25.0984	0.2120
119	Lak_24/R2	2.9607	-25.5015	0.4425
120	Lak_25/R2	3.3299	-25.1229	0.8323
